# ﻿Development ﻿﻿of﻿ ﻿﻿a facescan 3D facial reconstruction technology method for quantitative evaluation of cheilitis granulomatosa

**DOI:** 10.1038/s41598-017-01378-z

**Published:** 2017-05-02

**Authors:** Chang-Qing Mu, Shi-Qin Wang, Yang Liu, Chun-Lei Li, Xiao-Sheng Hu, Hong Hua

**Affiliations:** Department of Oral Medicine, Peking University School and Hospital of Stomatology & National Engineering Laboratory for Digital and Material Technology of Stomatology, 22 South Zhongguancun Ave, Haidian District Beijing, 100081 China

## Abstract

We explored the applicability of Facescan three-dimensional (3D) facial reconstruction technology for adjunctive diagnosis and therapeutic evaluation of cheilitis granulomatosa (CG) in 33 patients with CG and 29 healthy controls at the Dept. of Oral Medicine, Peking University, School and Hospital of Stomatology (PKUSS), from January 2015 to May 2016. The Facescan structured-light 3D facial reconstruction scanner was used to scan the scope of lips in both groups, in order to acquire 3D morphological data of the lips. The lengths of six characteristic line segments were measured from the 3D lip model of the two groups, and the acquired data were compared. The results showed that the distance between the labiale superius and labiale inferius, and the lengths of the upper and lower vermilion borders showed significant differences between the CG and control groups, by using the 3D lip model. Thus, Facescan 3D facial reconstruction technology showed good reproducibility in the evaluation of lip swelling in CG patients, and it can be used to analyse the degree of lip swelling and evaluate the therapeutic efficacy of different treatments for CG.

## Introduction

Cheilitis granulomatosa (CG), also known as Miescher cheilitis, is a chronic granulomatous disease characterized by repeated swelling of the lips and refractory lesions^1, 2^. Its main clinical presentation includes swelling of the lips, face, and/or gums^3, 4^. Current understanding on the aetiology and pathogenesis of CG are still incomplete^5, 6^. Some studies have shown that CG may be related to several factors such as infection, genetics, and allergic reactions^7, 8^. To date, standardized and curative treatment methods for CG are yet to be identified. Moreover, the evaluation of lip swelling and therapeutic efficacy of CG does not follow a standard criteria or achieve consensus. The evaluation of therapeutic effects across most studies has been mainly based on the patients’ self-perception and doctors’ subjective evaluation^9–11^. Objective criteria for evaluation of treatment are still lacking.

The morphological features of various facial diseases have always drawn attention in orthodontics, orthognathic surgery, prosthodontics, and plastic surgery^12, 13^. Dental practitioners have long focused their research on the measurement of human facial anatomical features^14–16^. In recent years, optical 3D measurement techniques have been extensively applied in dental areas^17^. The objective evaluation of facial morphology is the fast and accurate three-dimensional (3D) measurement of the real morphology of facial soft tissues. The 3D measurement technique is regarded as optical measurement for facial soft tissues, because of the high image resolution of data acquisition and the ability to add real-skin–texture information.

Optical 3D facial measurement techniques include line laser, stereoscopic photography, and structured-light technology^18^. The mainstream 3D scanners based on these three principles include Faro line laser scanners, 3D-MD stereoscopic photography, and Facescan structured-light scanners. Zhao *et al*. conducted a horizontal comparison of the imaging accuracy of three facial scanners based on different scanning principles^18–20^. Their research results indicated that there were no statistically significant differences between the measurement values and standard value for these three mainstream scanners. Furthermore, the measurement data of all three scanners had good reliability^21^.

This study applied 3D reconstruction technology to acquire and compare the 3D facial information of patients with CG and healthy controls with the aim of identifying characteristic landmarks that can reflect the 3D lip morphology of patients with CG. We hypothesized that our results may provide a preliminary confirmation for the characteristic 3D lip morphology of patients with CG and likely be considered as an objective index for the clinical diagnosis and evaluation of therapeutic efficacy in CG.

## Results

### Characteristics of the two study groups

The characteristics of both study groups is shown in Table 1. In the CG group, 33 patients were included. Among them, 14 had swelling of the upper lip, 14 had swelling of the lower lip, and 5 had swelling of both lips. The control group included 29 age- and gender-matched healthy subjects (16 male and 13 female; age range, 18–59 years).

**Table 1:** Baseline information of the CG and control groups.

	Number of subjects	Mean age (X ± S)	Gender		Swelling of lips		
			F	M	Lower lip	Upper lip	Both lips
CG group	33	42.28 ± 11.70	15	18	14	14	5
Control group	29	41.00 ± 12.04	13	16	0	0	0

### Repeatability test

Line data (Sn-Ls) were collected from the healthy control group for repeatability analysis. The measured mean values and multiple comparisons are shown in Tables 2 and 3. The statistical results of multiple comparisons showed no significant differences among the pairwise comparisons for the three sets of data (P > 0.05). Therefore, we concluded that the experimental repeatability was reliable.

**Table 2:** Measurements of Sn-Ls in the healthy control group.

No.	Gender	Age	Sn-Ls1	Sn-Ls2	Sn-Ls3	Mean
1	F	30	11.65	11.73	11.60	11.66
2	M	26	17.01	15.93	16.25	16.40
3	F	28	12.96	13.01	12.85	12.94
4	M	28	16.11	15.97	16.38	16.15
5	F	47	15.84	15.09	15.04	15.32
6	F	50	13.19	13.19	13.39	13.26
7	F	46	15.30	15.28	14.39	14.99
8	F	44	18.90	19.26	19.00	19.05
9	F	44	15.94	15.74	15.81	15.83
10	M	27	22.43	23.17	22.81	22.80
11	M	49	19.06	18.75	19.02	18.94
12	M	50	21.34	22.77	22.12	22.08
13	M	59	28.13	27.95	28.45	28.18
14	M	55	18.82	18.96	18.48	18.75
15	M	50	15.85	15.03	16.41	15.76
16	M	30	16.73	16.81	15.66	16.40
17	M	36	18.23	17.83	17.71	17.92
18	M	52	18.85	18.96	17.63	18.48
19	M	48	22.90	22.91	23.07	22.96
20	F	48	13.62	14.35	13.46	13.81
21	M	46	15.48	15.35	16.48	15.77
22	M	55	18.76	18.27	18.77	18.60
23	M	50	15.01	16.53	15.94	15.83
24	F	27	14.77	14.50	15.17	14.81
25	F	20	14.44	14.41	14.82	14.56
26	F	49	17.91	17.92	17.73	17.85
27	F	18	11.58	12.15	11.97	11.90
28	F	52	16.61	15.58	16.11	16.10
29	M	25	16.47	17.07	16.27	16.60

**Table 3:** Multiple comparisons among the mean values of the three measurements.

	Group	Group	Difference in means	Standard error	Significance	95% confidence interval	
						Lower limit	Upper limit
Sn-Ls	1	2	−0.02	0.93	0.98	−1.90	1.86
		3	0.04	0.94	0.97	−1.84	1.91
	2	1	0.02	0.94	0.98	−1.86	1.90
		3	0.06	0.94	0.95	−1.82	1.93
	3	1	−0.04	0.94	0.97	−1.91	1.84
		2	−0.06	0.94	0.95	−1.93	1.82

### Measurement results of Sn-Ls, Ls-Li, CphL-CphR, ChL-ChR, and length of vermilion border of the two groups

In the CG group, there were 19 cases of swelling of the upper lip (14 with swelling of the upper lip only +5 with swelling of both lips) and 19 cases of swelling of the lower lip (14 with swelling of the lower lip only +5 with swelling of both lips). Descriptive statistics of the line data from both groups were obtained. The data generally showed a normal distribution. Among the 6 indices compared between both groups, statistically significant differences were found with respect to the measurements of Ls-Li, length of upper vermilion border, and length of lower vermilion border (Table 4).

**Table 4:** Statistical values for the lengths of characteristic lines of the two groups.

	CG group (X ± s)	Control group (X ± s)	T value	P value
Sn-Ls	17.02 ± 3.58	17.16 ± 2.98	0.165	0.870
Ls-Li	15.87 ± 2.98	22.85 ± 5.59	6.238	0.000
CphL-CphR	12.94 ± 1.78	13.56 ± 2.66	1.075	0.287
ChL-ChR	51.05 ± 3.82	52.41 ± 4.76	1.227	0.225
Length of upper vermilion border	69.12 ± 5.50	75.24 ± 8.49	4.350	0.000
Length of lower vermilion border	63.78 ± 5.39	68.08 ± 9.50	4.503	0.000

### Three-dimensional lip measurements of patients with CG before and after treatment

**Figure 4:**
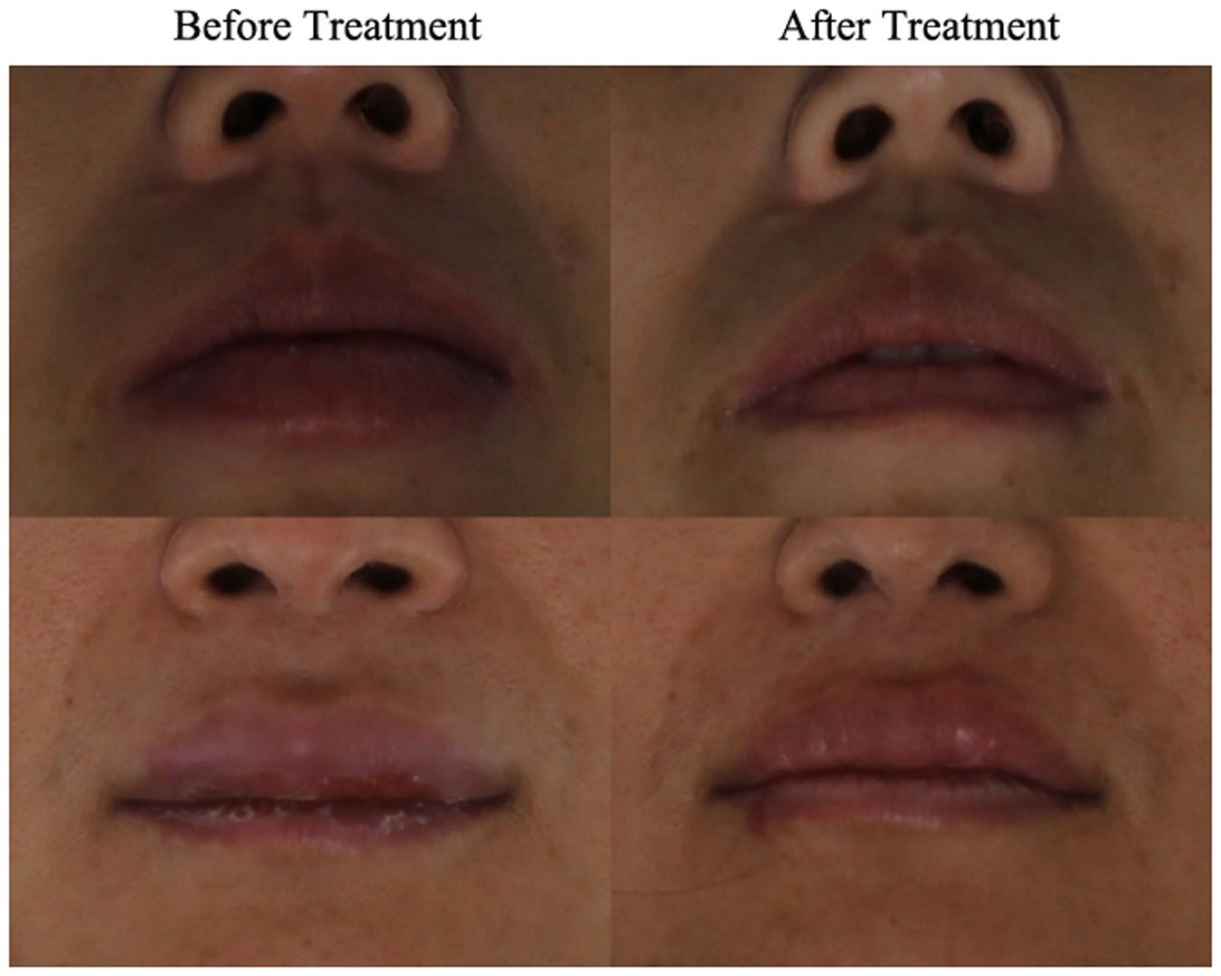
Lip changes before and after treatment.

**Figure 5:**
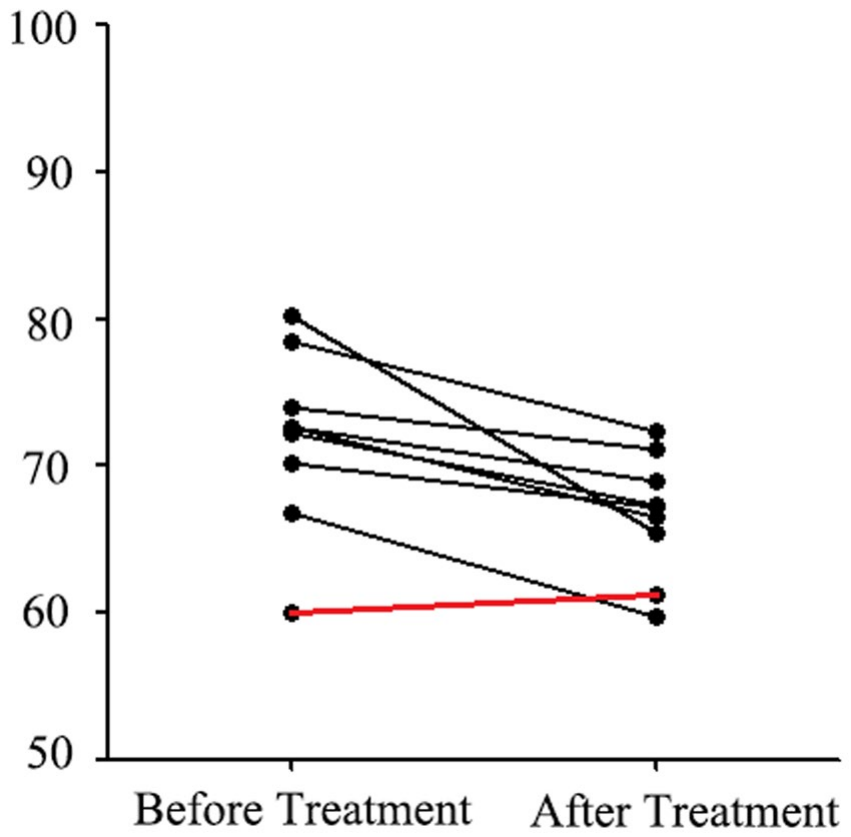
Trend of vermilion border lengths before and after treatment.

Independent samples *t*-tests results showed that the indicator reflective of lip thickness—Ls-Li—showed a significant difference before and after treatment (P = 0.000). The test also showed a significant difference in vermilion border length before and after treatment (P = 0.000). These results indicated that the vermilion border length showed downward trends after treatment. Patients’ lip changes before and after treatment are shown in Figs 4 and 5.

## Discussion

Cheilitis granulomatosa (CG) is a chronic granulomatous disease characterized by repeated swelling of the lips. Its clinical presentations include local swelling that feels soft and rubber-like, no pain or itching, and non-pitting oedema under pressure^22^. This disease reportedly shows no significant biases with regard to gender or age, and has a low incidence in the general population. Studies have indicated that the annual incidence of CG is 0.3–8/100,000^23^, and past reports on CG have been relatively rare. At present, the aetiology and pathogenesis of CG are still unclear^24^. Swelling of the lips is a typical symptom of CG, but there is still a lack of objective indicators to measure its degree, assess grading, and determine the severity. So far, there is no quantitative method to evaluate lip swelling in patients with CG. Moreover, the evaluation of therapeutic efficacy of CG does not have any standard criteria or achieve consensus. We believe that this study provides an objective method for this purpose. We used Facescan 3D facial reconstruction technology to evaluate the degree of lip swelling in CG patients, and assess whether the 3D Scan could be used as an objective indicator to evaluate therapeutic efficacy.

The lip landmarks selected in this study are consistent with those used in other similar studies on maxillofacial symmetry^25, 26^. However, thus far, no studies have referred to the 3D Scanner being applied in mucosal disease or CG. Hence, we consider this study to be highly representative. Furthermore, the results of consistency tests performed prior to the formal experiment indicated that the measurement methods used in this study had good reliability. The six sets of line data measured and compared between the healthy control and CG groups showed significant differences with respect to Ls-Li and the lengths of the upper and lower vermilion borders. The three remaining sets of data (sn-Ls, CphL-CphR, and ChL-ChR) did not show such significant differences. Therefore, our results indicate that Ls-Li and the lengths of the upper and lower vermilion borders can be used as objective indicators for the degree of lip swelling in CG. This can serve as an objective index for the clinical assessment of degree of lip swelling. The acquisition process of the facial morphological data via Facescan 3D facial reconstruction technology was simple, quick, and had a high level of accuracy^27^. Hence, this method could meet the needs of clinical diagnosis as a simple and objective diagnostic method for assessing the degree of lip swelling.

To date, effective treatment methods for CG are still lacking^6, 28–31^. The treatment methods that are commonly employed in clinics include local injection of glucocorticoids; subcutaneous injection of adalimumab; and oral doses of metronidazole, roxithromycin, clofazimine, or dapsone. Triamcinolone acetonide (TA), a long-acting glucocorticoid, is the most commonly used drug to treat CG. Its specific mechanisms in the treatment of CG might be related to the reduction of local inflammatory reactions and interstitial oedema^31^. We used the 3D Facescan to evaluate the therapeutic efficacies for CG, and significant differences were found in Ls-Li and the vermilion border length before and after treatment with TA; whereas, the other characteristic line data were not found to be significantly different after treatment. The results showed that TA local injection was an effective treatment for CG. The thickness of both lips and the vermilion border lengths of the affected lip showed differences before and after local injections of TA and can be used as an objective parameter for evaluating the effectiveness of therapy. In addition, we found that the vermilion border lengths of the patients showed a decreasing trend. Furthermore, clinical examination revealed that the degree of lip swelling reduced and the texture became soft after treatment.

This study had some limitations. Owing to the low incidence of CG in the general population, our sample size was relatively small. Future studies in larger cohorts will likely enable a more accurate understanding and analysis on the characteristics of lip swelling in patients with CG. In addition, the observation period should be extended in order to more accurately evaluate the therapeutic efficacy of different drugs via the 3D scan.

To our knowledge, this is the first study to use Facescan 3D facial reconstruction technology to objectively evaluate the degree of lip swelling in CG. The preliminary results showed that 3D scan technology had good repeatability in the evaluation of lip swelling in patients with CG. Ls-Li and the lengths of the upper and lower vermilion borders can be used to evaluate the degree of lip swelling in CG. The data of lip morphology via 3D Facescan can be used as objective indicators for the adjunctive diagnosis of the degree of lip swelling and the evaluation of therapeutic efficacy of CG.

## Materials and Methods

### Research Design

This case-control study was approved by the institutional review board of Peking University School of Stomatology (PKUSSIRB-201520016). ﻿Written informed consent ﻿were obtained from all subjects before the study.

#### Case group

33 patients confirmed as CG by clinical and pathological examination, who were admitted to the Dept. of Oral Medicine, Peking University School and Hospital of Stomatology (PKUSS) from January 2015 to May 2016, were included in this study. Among them, 8 patients were treated with local injection of triamcinolone acetonide (TA), 2–4 times after establishing the diagnosis.

#### Control group

29 age- and gender-matched healthy volunteers were enrolled in the study during the same study period.

### Inclusion criteria for CG patients

Patients in the age range of 10–70 years who fulfilled the clinical and pathological diagnostic criteria for CG and without mental disorders, sarcoidosis, Crohn’s disease, tuberculosis, and possessing both autonomous behaviour and decision-making capacity were included in this study. We had no restrictions on gender or ethnicity.

### Exclusion criteria for CG patients

Patients who did not agree to participate in this study or were under long-term, systemic glucocorticoid treatment due to other diseases or received local or systemic glucocorticoid therapy within 1 month before admission were excluded. In addition, patients having severe cardiovascular diseases or acute, systemic infectious diseases were also excuded.

### Experimental equipment and software

**Figure 1:**
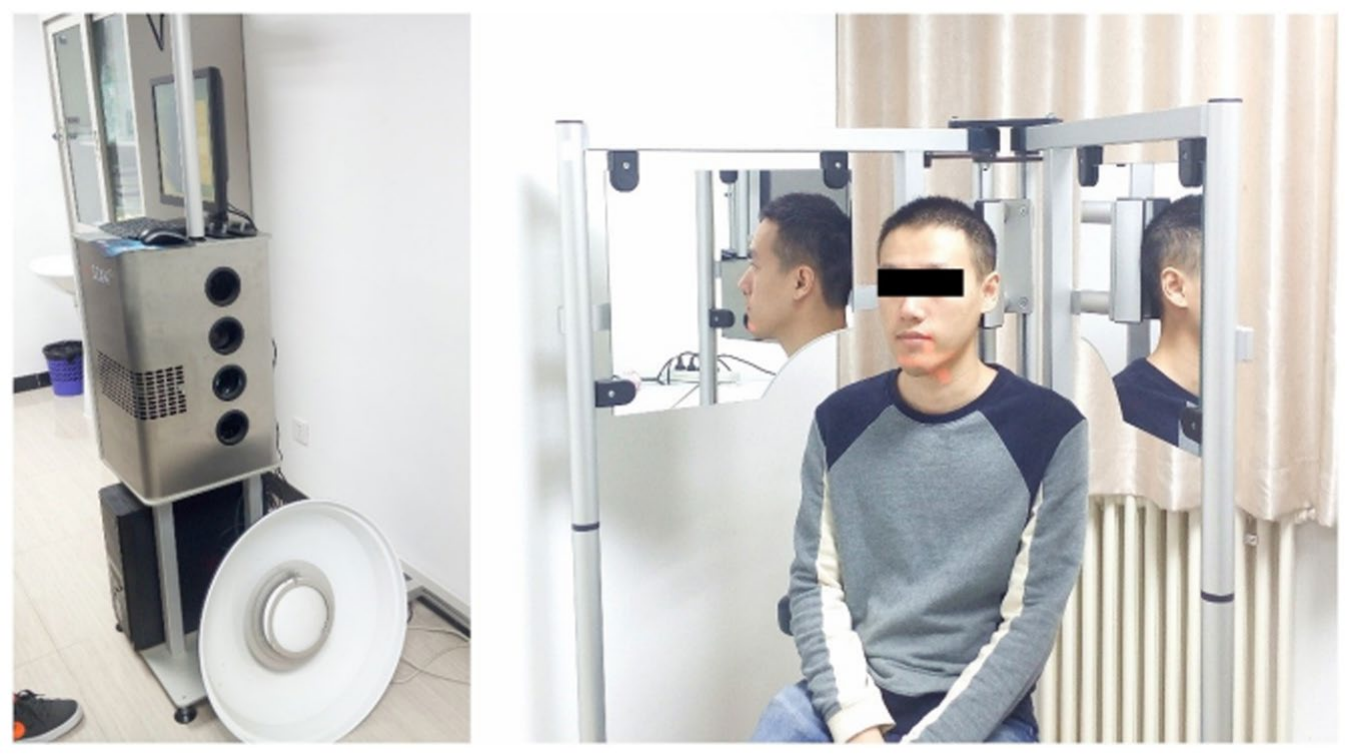
Facescan structured-light 3D scanner.

Facescan structured-light 3D scanner (Fig. 1) was purchased from 3D Systems GmbH, Germany; its theoretical scanning accuracy was 0.1 mm. The principle of this scanner involves projecting structured-light from a light source onto the target surface, which generates deformations in the structured-light. The patterns of the deformed structured-light are then captured by a camera for analysis. Then, the corresponding 3D reconstruction algorithm is implemented, in order to obtain the 3D measurement of the target surface morphology.

The software used in this study included: 3D reverse engineering software, Geomagic Studio 12; electronic spreadsheet, Microsoft Excel 2013; and statistical analysis software package, SPSS 22.0.

### Selection of characteristic landmarks

**Figure 2:**
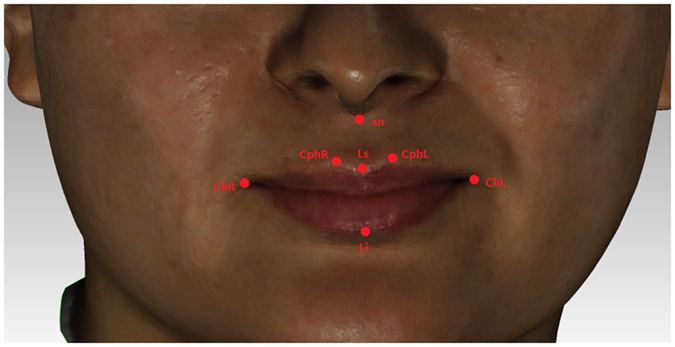
Selection of facial landmarks.

Subnasale: sn; Labiale superius: Ls; Labiale inferius: Li; Crista philtri Left/Right: Cph L/R; Cheilion Left/Right: Ch L/R (Fig. 2).

### Measurement of lip data

Line data: Sub nasale–labiale superius: sn-Ls; labiale superius–labiale inferius: Ls-Li; distance between the left and right crista philtra: CphL-CphR; width of labial fissure: ChL-ChR; length of upper vermilion border; length of lower vermilion border. ﻿

Data acquisition were conducted by Dr.Mu and/or Wang.﻿ Before the study, Dr.Mu and Wang were trained by professional worker in Lab for Digital and Material technology of PKUSS. ﻿The length ﻿of each characteristic line was measured in triplicate by two independent researchers, using Geomagic Studio with reliable consistency (Kappa value: 0.87).

The characteristic facial landmarks of the subjects in the CG and control groups were labelled according to the above methods, and the relevant data measurements were performed.

### ﻿﻿﻿Measurement of characteristic facial morphology in CG patients before and after TA treatment via the Facescan structured-light 3D scanner

**Figure 3:**
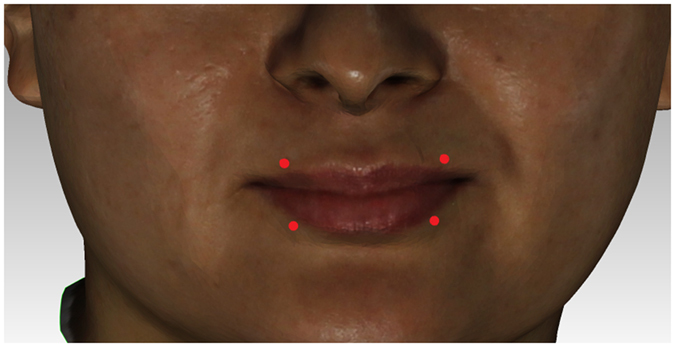
Schematic diagram of injection sites.

Eight patients with CG, after diagnosis, received local injections of TA hormone (40 mg: 1 mL) + 2% lidocaine (100 mg: 5 mL). A 1-mL mixture was then injected into the lesion site of the lip, at a dose of 0.5 mL per site (Fig. 3)^32, 33^. Three local injections were administered at 0 wk, 1 wk, and 2 wk after the diagnosis was confirmed. Tre﻿atment Evaluation for CG patients via the Facescan structured-light 3D scanner.

Then, the Facescan structured-light 3D scanner was used to acquire characteristic 3D facial data in order to compare the effectiveness before and after treatment.

### Statistical analysis and methods

The values were recorded in an Excel spreadsheet, and the mean value was calculated. The lengths of the upper and lower vermilion borders were measured after segmentation and projection using Geomagic Studio, and the respective sum totals were obtained. The measurement was repeated three times, and the mean value was recorded.

SPSS was used to calculate the mean values of the data from the two groups. The basic information and each set of line data were compared between the experimental and control groups using *t*-tests. The differences before and after treatment among CG patients were compared using paired-samples *t*-test. A significance level of P < 0.05 indicated that the difference was statistically significant.

### Ethical approval and consent to pa﻿rticipate

This case-control study was approved by the institutional review board of Peking University School of Stomatology (PKUSSIRB-201520016). The methods used in this study were carried out in accordance with ethical principles of research. And informed consent was obtained from all subjects.
